# Supplementary figures and images for: AutoNeuriteJ: An ImageJ plugin for measurement and classification of neuritic extensions
Source: PLoS One. 2020 Jul 16;15(7):e0234529. doi: 10.1371/journal.pone.0234529 (PMC7365462; doi:10.1371/journal.pone.0234529)

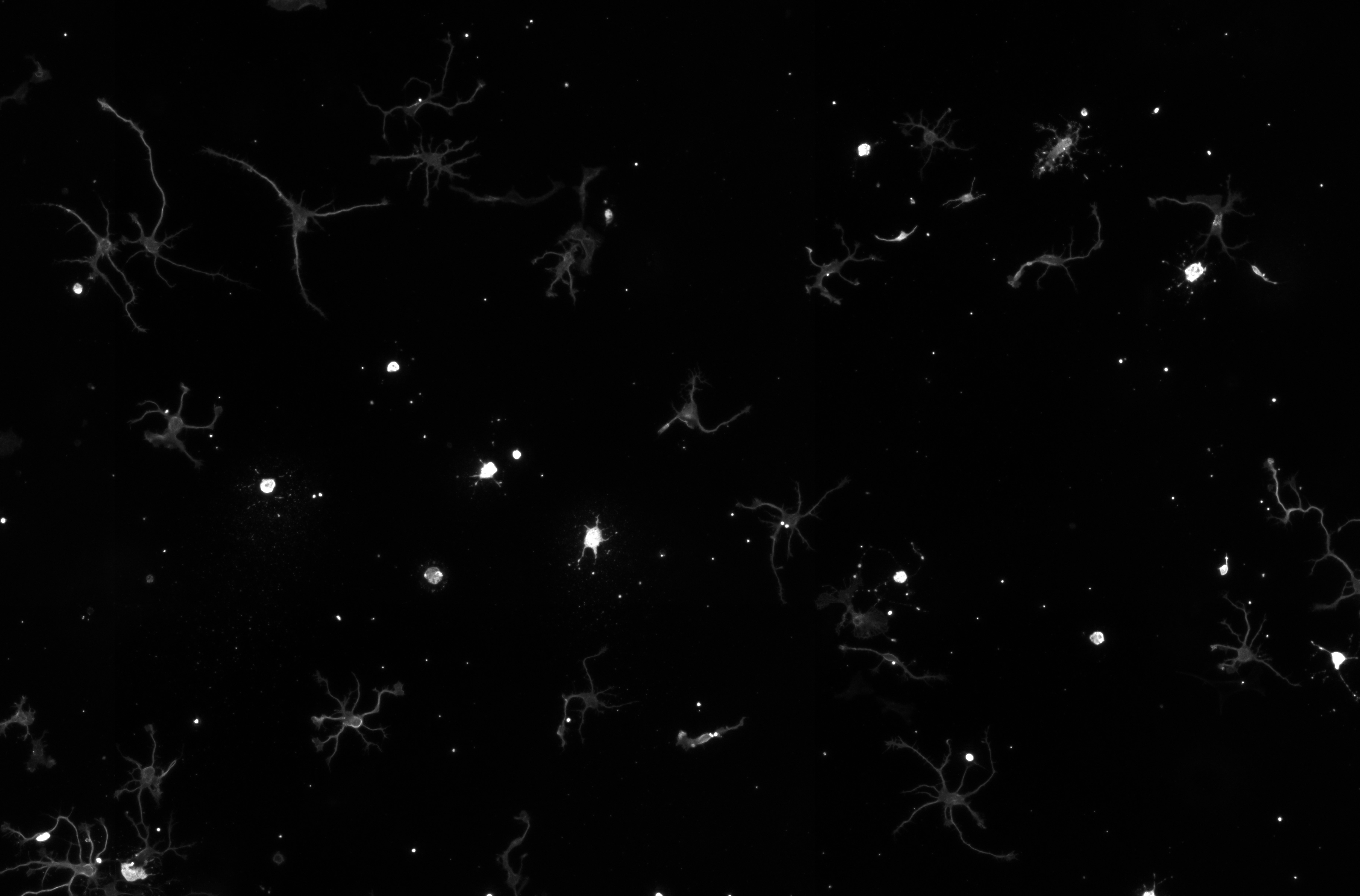

Supplement: S1 Data — (ZIP) [file pone.0234529.s001.zip › AutoNeuriteJ-master/Demo-Image_Neurons_Cy3.tif]

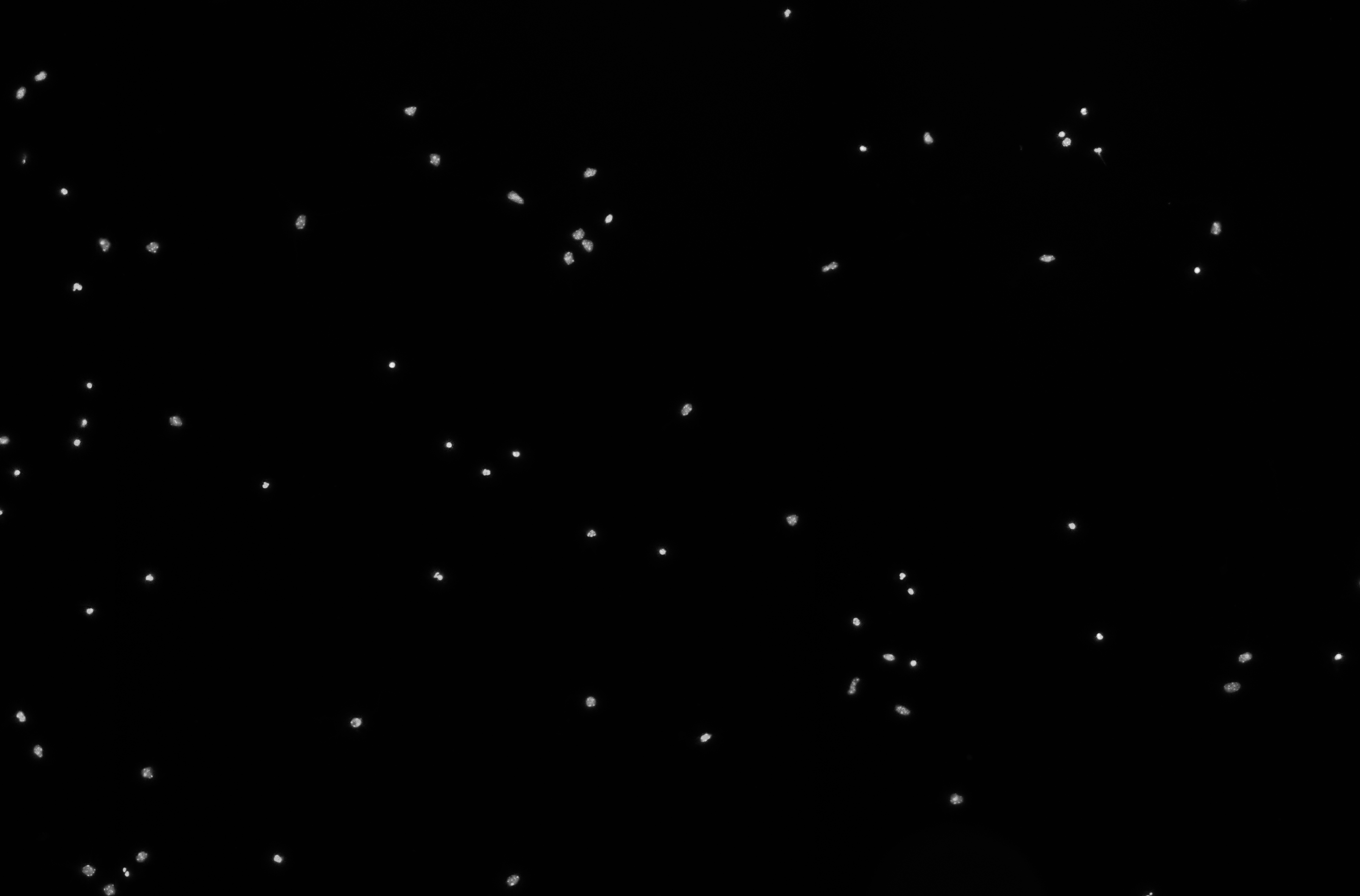

Supplement: S1 Data — (ZIP) [file pone.0234529.s001.zip › AutoNeuriteJ-master/Demo-Image_Nuclei_DAPI.tif]
