# Supplementary material for: AutoNeuriteJ: An ImageJ plugin for measurement and classification of neuritic extensions
Source: PLoS One. 2020 Jul 16;15(7):e0234529. doi: 10.1371/journal.pone.0234529 (PMC7365462; doi:10.1371/journal.pone.0234529)
Supplement: Attachment — Submitted filename: ResponseToReviewer.docx [file pone.0234529.s003.docx]

Dear Editor,

We thank the reviewer and the editor for their interest in our work.

We corrected the manuscript according to the reviewer's recommendations. We hope that our manuscript is now acceptable for publication in Plos One.

Response to the reviewer :

"1. Please briefly describe the criteria for indicating if a neuron possesses an axon/is polarized. This information could be presented in Part III: Measurement and classification of neurites"

We clarified the criteria used in our study in the text :

The longest neurite will be considered as an axon if it satisfies to standard criteria [11], *i.e.* in our study the criteria were set to : the length of the axon should be twice the size of the second longest neurite and longer than 100 pixels . These parameters can be set by the user.

"2. In Figure 3A, the biggest outliers are all above the line, indicating that when analyzed by AutoNeuriteJ, these outliers have ~10-70% greater lengths as estimated by eye from Figure 3A This result suggests that the outliers are not random but may be due to some systematic difference in the measurement process for about 5% of the neurons. This is not a serious issue but if the authors have some insights into how these discrepancies could arise, it would be very useful to discuss this briefly since it can help users understand how to optimize their analysis."

We obtained insight for the outliers and added theses informations in the text.

By inspecting the data corresponding to points far above the fitted line we observed that they represent cells that have not been segmented correctly (e.g. Several cells are fused and present a single nucleus). These cells can be easily removed from the binarized stacks of neurons before the neurite measurement part of AutoNeuriteJ.

"3. There are some minor typos and phrases that could be tweaked for better clarity:- Figure 5 legend: "Length of the longest neurites for cells presenting an axon or not treated with Sema3E" should be rephrased or have a comma after "not" to remove ambiguity and make clear that the groups are not "presenting an axon" and "not treated with Sema3E."

The sentence has been rewritten to : (A) Length of the longest neurites for polarized and unpolarized cells treated or not with Sema3E

"4- Page 8, paragraph 2: "the mean length of primary neurites without the longest in unpolarized cells" - this phrase needs a noun after the word "longest."- Page 12, paragraph 1: "wiskers" is a typo - should be "whiskers"

The manuscript has been corrected .
